# Robust Assessment of Homologous Recombination Deficiency Genomic Instability by OncoScan Microarrays

**DOI:** 10.1016/j.jmoldx.2025.02.011

**Published:** 2025-04-04

**Authors:** Ariadna Lara Gutierrez, Iris Halbwedl, Stefan Sauer, Peter Regitnig, Edgar Petru, Rita Seeböck, Susanne Schubert, Cornelia Peternell, Koppány Bodó, Kurt Prein, Karl Kashofer

**Affiliations:** ∗ Diagnostic and Research Institute of Pathology, Medical University of Graz, Graz, Austria; † Division of Gynaecology, Medical University of Graz, Graz, Austria; ‡ Institute of Clinical Pathology and Molecular Pathology of the Lower Austria Central Region (University Hospital St. Pölten), Karl Landsteiner University of Health Sciences, Krems, Austria; § Division of Gynaecology and Obstetrics, University Hospital St. Pölten, St. Pölten, Austria; ¶ Karl Landsteiner University of Health Sciences, Krems, Austria; || Division of Gynaecology and Obstetrics, Hospital Hochsteiermark, Leoben, Austria; ∗∗ Institute of Pathology, Hospital Hochsteiermark, Leoben, Austria

## Abstract

Genomic instability scars are markers for detecting homologous recombination deficiency (HRD) status in patients with ovarian cancer and predicting the response to poly (ADP-ribose) polymerase inhibitor treatment. Currently, only a few reliable and validated assays are available, with the Myriad myChoice CDx being the most commonly used commercial assay for genomic instability scar score determination. Given the need for a more straightforward, accessible, and reliable method for detecting genomic instability scars methods, in this work, we describe the feasibility of using the microarray OncoScan copy number variant assay and open-source software packages to quantify genomic instability scores, and the development of an open-access online platform for genomic instability score calculation. The laboratory-developed test accurately classified homologous recombination–proficient and recombination–deficient samples based on genomic instability scores derived from the OncoScan copy number variant assay. Internally evaluated genomic instability scores demonstrated a 92% overall agreement and a higher sample success rate compared with externally analyzed genomic instability scar scores. The availability of HRD determination has doubled the number of patients eligible for poly (ADP-ribose) polymerase therapy. The assay can be conveniently performed on individual samples, and the open-access online platform facilitates HRD determination without the need for specialized bioinformatics support.

Ovarian cancer is the eighth most common cancer among women worldwide, with >310,000 new cases reported in 2022 (6.6/100,000 incidence rate). It ranks seventh in cancer-related mortality among women (4.2/100,000), with a 5-year survival rate ranging from 36% to 49%.^1^ In Austria, the average annual incidence of ovarian cancer from 2018 to 2022 was 716.3 cases, representing 4% of diagnosed tumors. During the same period, the average annual mortality was 500 cases (Statistics Austria, *https://www.statistik.at/en/statistics/population-and-society/health/cancer*, last accessed February 15, 2023). Studies indicate that women with a family history of ovarian cancer have a significantly higher risk of developing the disease, with pathogenic germline variants in the *BRCA1/2* genes accounting for a substantial proportion of cases.^2^ These variants increase the risk of developing ovarian cancer by up to 60% compared with the general population.^2^^,^^3^

Bi-allelic variants in *BRCA1* or *BRCA2* genes disrupt the homologous recombination repair mechanism, preventing high-fidelity double-stranded break repair, which can lead to aberrant cell proliferation.^4^ Homologous recombination deficiency (HRD) associated with the *BRCA1/2* pathogenic variants is present only in 30% of the ovarian cancer tumors, meaning the remaining 70% are attributable to other genes.^5^^,^^6^ The homologous recombination pathway involves >30 genes, and pathogenic variants in any of these genes can impair the homologous recombination repair mechanism. The large number of genes and the challenge of assigning pathogenicity to single-nucleotide variants make it difficult to assess HRD status based solely on mutations in HRD genes.

Alternatively, HRD determination is performed by analyzing patterns of acquired numerical and structural chromosomal abnormalities, collectively referred to as genomic instability scars (GISs).^7^ Common GIS patterns include loss of heterozygosity,^8^ large-scale state transitions,^9^ and telomeric allelic imbalance.^10^ In the United States, the US Food and Drug Administration has approved commercial HRD companion diagnostics, such as those performed by Myriad myChoice CDx (Salt Lake City, UT) and FoundationOne CDx (Foundation Medicine, Boston, MA).^11^ Additionally, complementary laboratory-developed test (LDT) platforms using single-nucleotide polymorphism arrays are available for determining HRD status based on genomic instability.^12^ In the European Union, the European Medicines Agency permits GIS (loss of heterozygosity, large-scale state transition, and telomeric allelic imbalance) scoring to be performed by accredited laboratories using validated tests.^4^

HRD determination is of great significance for patients with HRD tumors, as they can benefit from poly (ADP-ribose) polymerase (PARP) inhibitor therapies. PARP inhibitors prevent the repair of single-stranded breaks, which are common lesions induced by environmental factors or platinum-based chemotherapy.^13^ In the absence of efficient single-stranded break repair, many of these breaks progress to double-stranded breaks that cannot be repaired in HR-deficient tumors. This leads to synthetic lethality by forcing cells to frequently use error-prone nonhomologous end-joining repair, which leads to cell death.^13^ HRD tumors can benefit from PARP inhibitor therapies even without *BRCA1/2* pathogenic variants, as demonstrated by the PAOLA-1 (Platine, Avastin and OLAparib in 1st Line) study, in which patients without *BRCA1/2* pathogenic variants but with high GIS scores showed increased overall progression-free survival for up to 11 months.^5^ The European Medicines Agency initially approved the PARP inhibitor olaparib as a second-line maintenance treatment in December 2014.^11^^,^^14^ Since then, two more PARP inhibitors have been approved: niraparib (regardless of the status of the *BRCA1/2* variants) in November 2017 and rucaparib (as rescue therapy) in May 2018.^11^^,^^14^

Given the importance of determining HRD and the incidence of ovarian cancer in the Austrian population, it is imperative to perform local diagnostic tests in European clinical laboratories. There is a growing need to establish and validate easily accessible methods for determining HRD. In this study, the development of a GIS scoring method based on OncoScan microarrays, its validation against Myriad myChoice CDx, and the implementation of an online platform for hands-free HRD determination are described. It is demonstrated that local methods can be applied and automated to correctly classify HRD-positive and HRD-negative samples based on the genomic instability score.

## Materials and Methods

### Ethical Approval

HRD-GIS and *BRCA1/2* variant results from patients were collected according to ethics approval number 35-274 ex 22/33 issued by the Ethics Commission of the Medical University of Graz (Graz, Austria).

Paraffin-embedded specimens were collected according to ethics approval number 33-113 ex 20/21. All the patients provided written informed consent to participate in the study.

### Sample Collection

This study included formalin-fixed, paraffin-embedded (FFPE) samples from patients diagnosed with ovarian cancer collected over a 28-month period. The samples were divided into two sets: set I: 730 samples analyzed for *BRCA1/2* pathogenic variants and homologous recombination deficiency by Myriad myChoice CDx; and set II: 286 samples analyzed at the Medical University of Graz.

### External HRD Determination

External determination of homologous recombination deficiency status was performed using Myriad myChoice CDx. The GIS score considers loss of heterozygosity, telomeric allelic imbalance, and large-scale state transitions. Additionally, the status of *BRCA*1/2 variants was analyzed and reported as positive or negative.

### Pathogenic Variants of *BRCA1* and *BRCA2* Genes

Myriad myChoice CDx considers *BRCA1* or *BRCA2* to be altered when deleterious or suspected deleterious variants are present, such as nonsense and missense mutations [eg, *BRCA1* (NM_007294.4; VarSome, *https://varsome.com*, last accessed June 7, 2024) c.181T > G (p.Cys61Gly)].

In-house analysis of *BRCA1/2* variants was performed using the Ion Torrent Oncomine BRCA1&2 Panel (Thermo Fisher Scientific, Waltham, MA), according to the manufacturer's instructions. Briefly, 10 ng of DNA was used as the input, amplified with *BRCA1/2* primers, followed by primer digestion, barcode addition, and amplicon purification. Finally, the libraries were quantified and loaded onto a chip for sequencing. This method allowed analysis of the entire coding sequences of *BRCA1* and *BRCA2*. The identified variants were annotated using VarSome,^15^ which provides pathogenicity classification criteria according to the Evidence-based Network for the Interpretation of Germline Mutant Alleles (ENIGMA) variant interpretation guidelines.

Quality assessment of the Oncomine *BRCA1/2* analysis included ensuring a minimum of 70% of the expected reads (number of amplicons × read depth), coverage overview, base-pair length, Phred-score plot, end-to-end reads, deamination index <10, and balanced primer pool distribution.

### Laboratory-Developed Test for GIS Scoring

The LDT for GIS scoring was performed on FFPE samples with a minimal tumor content of 20%, as determined by in-house pathologists (including P.R.). A minimum of 80 ng of the extracted DNA was required for testing. The OncoScan CNV Plus Assay (Thermo Fisher Scientific; catalog number 902694) was used according to the manufacturer's instructions. Briefly, DNA was annealed with the probe panel, followed by probe linearization, digestion, and overnight hybridization of the micro-arrays at 49°C. The next day, the arrays were washed and scanned using the GeneChip Scanner 3000 7 G (Thermo Fisher Scientific). Quality assessment of the OncoScan copy number variant (CNV) analysis included the following: quality control gels for size distribution and proper digestion control, visual inspection of homogeneous chip intensity, median of the absolute values of all pairwise differences ≤ 0.30, and single-nucleotide polymorphism quality control of normal diploid markers ≥ 26.

A computational analysis of the raw data files (.CEL files) was performed using R software version 3.6.1 (*https://www.r-project.org*). The analysis pipeline incorporated several open-source packages combined with a custom-made script to allow smooth output-input interaction. The packages used included Easy Copy Number (*https://github.com/gustaveroussy/EaCoN*) for normalization and segmentation; Allele-Specific Copy Number Analysis of Tumors^16^^,^^17^ for window segmentation, ploidy, and allele-specific copy number profiles; and scarHRD^18^ for the determination of genomic instability scars. These genomic instability scars include telomeric allelic imbalance (number of regions with allelic imbalance that extend to the telomeric region), large-scale state transition (number of chromosomal breaks with a minimum size of 10 megabases and a distance between these breaks of 3 megabases), and loss of heterozygosity (number of regions with any type of loss of heterozygosity >15 megabases). The use of single-nucleotide polymorphism arrays also allows for the assessment of copy number neutrality. The sum of the scars constituted the final GIS score.

The master function requires the user to provide the raw AT and GC CEL files, as well as the sample's sex specified as XX or XY. Certain default values are set, including AffyOncoScan as the analysis platform and penalty value of 70 for the Allele-Specific Copy Number Analysis of Tumors. Additionally, the reference genome hg19 is used for scarHRD. Further details are available on GitHub (*https://github.com/AriadnaLG/OpenHRD*).

A subgroup (*n* = 47) of samples analyzed with Myriad myChoice CDx with *BRCA1/2*-positive and *BRCA1/2*-negative results was stratified and randomized into training (*n* = 22) and validation (*n* = 25) cohorts. These groups were used to validate the proposed pipeline. Linear regression analysis was applied to the training group to align the in-house GIS scores from each sample with the scores generated by the Myriad myChoice CDx.

### Statistical Analysis

The correlation coefficient was calculated to compare the GIS scores between the Myriad myChoice CDx and LDT. Positive percentage agreement, negative percentage agreement, and overall percentage agreement were calculated to evaluate the performance of the LDT compared with the Myriad myChoice CDx.

### Clinical Data

Treatment decisions were requested and classified as platinum-based chemotherapy (PBC) alone, PBC/olaparib, or PBC/niraparib.

### Online Platform Implementation

To automatically calculate the GIS score, an R script version 3.6.1 was implemented to be used online using the Django Python Framework and Celery Task Queue. This script is available on GitHub (*https://github.com/AriadnaLG/OpenHRD*) and is described in Laboratory-Developed Test for HRD Scoring.

## Results

### Cohorts

In total, 730 FFPE samples with a tumor cell content >20% were shipped to Myriad for analysis. Of these, 14 (1.90%) were rejected and not analyzed by Myriad. Of the analyzed samples, 62 (8.49%) did not generate a GIS score, and the analysis of *BRCA1/2* variants in nine samples (1.23%) was not possible. This resulted in 645 samples (88.36%) with successful GIS and *BRCA1/2* variant analysis and 654 samples (89.59%) with successful GIS score analyses only. The age of the patients ranged from 27 to 100 years, with a mean age of 65 years.

**Figure 1:**
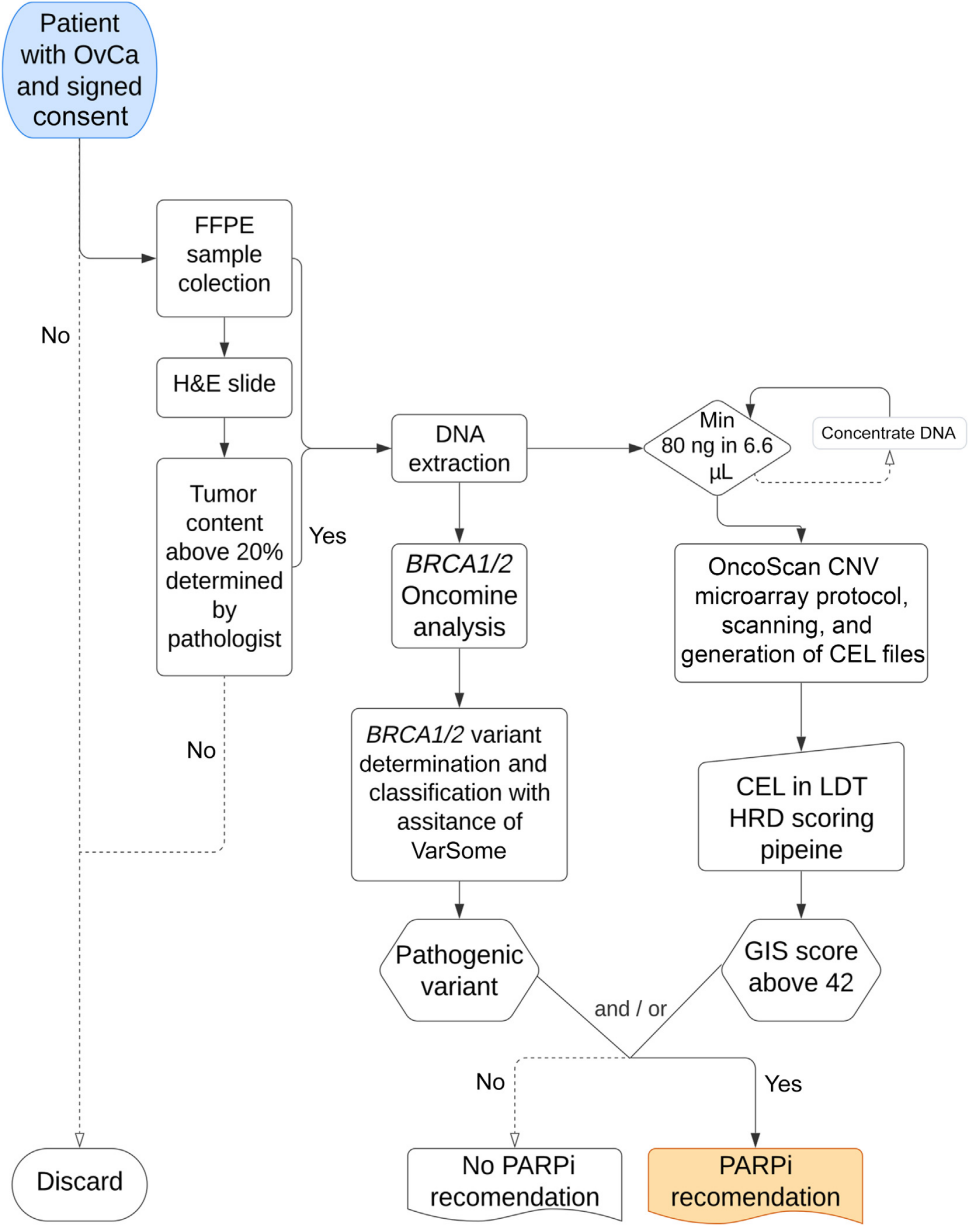
Diagram of laboratory-developed test (LDT) process applied to the samples. CNV, copy number variant; FFPE, formalin fixed, paraffin embedded; GIS, genomic instability scar; H&E, hematoxylin and eosin; HRD, homologous recombination deficiency; Min, minimum; OvCa, ovarian cancer; PARPi, poly (ADP-ribose) polymerase inhibitor.

For the internal analysis, samples with consent and a tumor cell content of a minimum 20% were accepted for the LDT analysis. A further description of the inclusion process is shown in Figure 1.

Of the 286 samples, 280 (97.90%) were successfully analyzed with GIS score determination. No *BRCA1/2* analyses were performed for the 12 externally collected samples. Six samples (2.10%) did not generate a valid GIS score because of poor DNA quality. The age of the patients ranged from 24 to 93 years, with a mean age of 65 years.

### Laboratory-Developed Test for HRD Scoring

**Figure 2:**
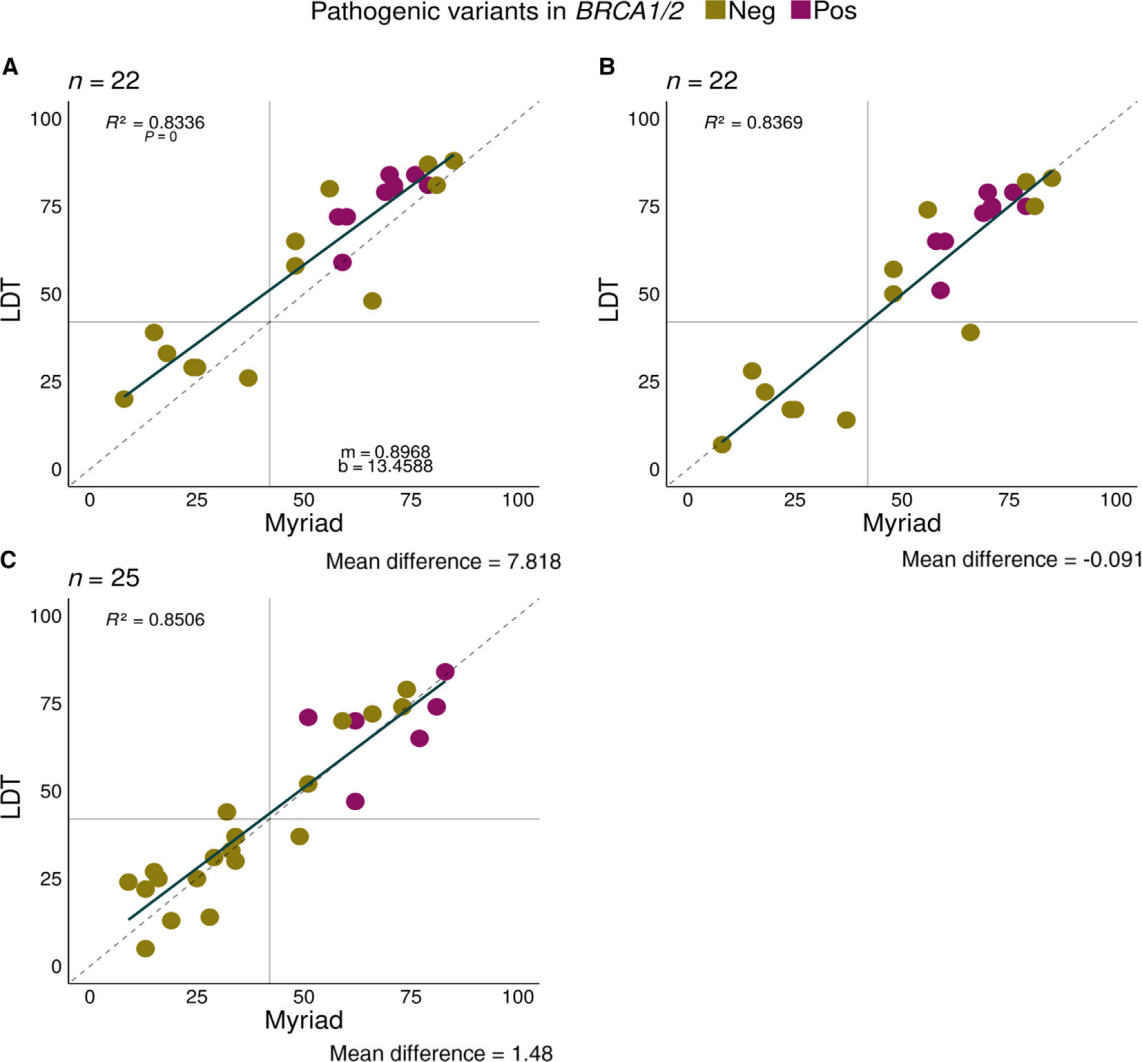
**A**–**C:** Comparison of Myriad myChoice CDx and laboratory-developed test (LDT) genomic instability score analysis results. Pathogenic *BRCA1/2* variants in violet, and nonpathogenic or non-*BRCA1/2* variants in green. **A** and **B:** Genomic instability scar (GIS) scores from 22 samples corresponding to the training group without (**A**) and with (**B**) GIS-corrected scores based on linear regression analysis correlation value of 0.83. Linear regression model provided the correction formula, where m represents the slope and b represents the value at which the *y* axis is intercepted. **C:** Validation cohort with applied correction, with correlation value of 0.85. In both training and validation groups, all cases with pathogenic *BRCA1/2* variants have positive GIS score (≥42). Neg, negative; Pos, positive.

Internal research from the training cohort (*n* = 22) showed a high correlation coefficient (0.83 among the Myriad results, *P* = 3.122 × 10^−9^. Using the clinically accepted threshold of 42 for ovarian cancer, all samples were classified into the same group using the LDT and Myriad assay (Figure 2A). In addition, all samples with pathogenic *BRCA1/2* variants [9 of 22 (41%)] showed a positive GIS score, whereas no or nonpathogenic *BRCA1/2* variants were detected in GIS-negative samples. However, the individual GIS score results showed an average difference of 7.8181 GIS points, with the difference being more pronounced in the lower range of GIS scores.

A linear regression analysis model was generated to improve the correlation with the Myriad results and correctly apply a threshold of 42 points for GIS-HRD determination. Linear regression was then applied to the same training cohort, reducing the mean difference between the samples to –0.091 GIS points. This application of the regression model led to misclassification of one sample (Figure 2B).

To evaluate the effectiveness of linear regression with respect to GIS score determination, a validation cohort of 25 independent samples was used, for which Myriad myChoice CDx results were also available. Like the training cohort, all *BRCA1/2* mutated samples (6 of 12) were observed to have a positive genomic instability score; these represent 50% of all GIS-positive samples (Figure 2C). After applying the linear correction factor, the sample scores showed an average difference of 1.48 GIS points and a correlation coefficient of 0.85 between the two groups. Two samples (8%) with GIS values close to the threshold were classified differently when compared with their Myriad myChoice CDx results.

The overall percentage agreement between Myriad myChoice CDx and LDT for the 47 samples was 93.6% (95% CI, 82.8%–97.8%), with a positive percentage agreement of 92.9% (95% CI, 77.4%–98.0%) and a negative percentage agreement of 94.7% (95% CI, 75.4%–99.1%). The distribution of positive and negative cases between the Myriad myChoice CDx and LDT is shown in Table 1.

**Table 1:** Contingency Table from the 47 Samples Analyzed with Myriad myChoice CDx and LDT

LDT	Myriad		
	Positive	Negative	Total
Positive	26	1	27
Negative	2	18	20
Total	28	19	47

LDT, laboratory-developed test.

**Figure 3:**
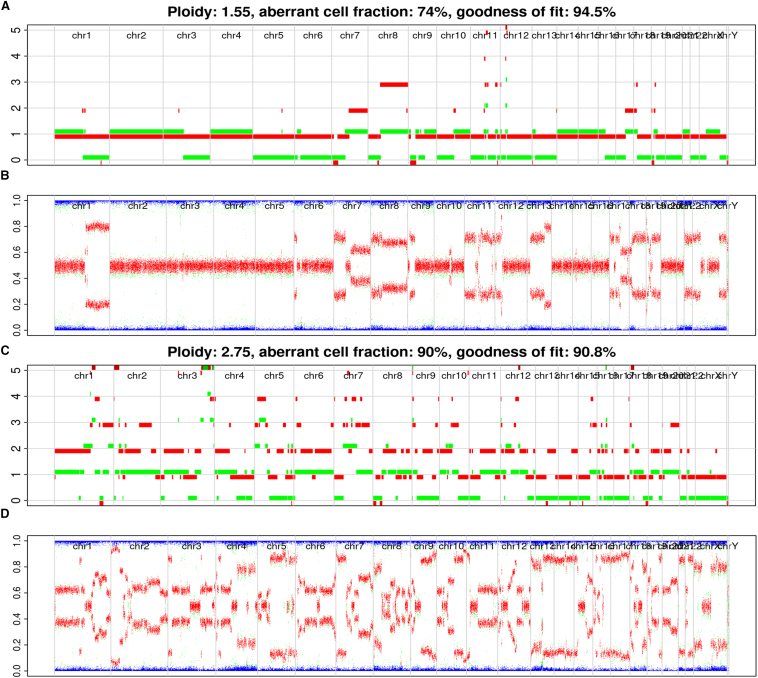
**A–D:** Allele-specific copy number and B-allelic frequency (BAF) plots from samples with low genomic instability scar (GIS) score (<42; **A** and **B**) and high GIS score (≥42; **C** and **D**). **A** and **B:** Allele-specific copy number plots where the major copy number is represented in red and minor copy number in green. **A:** Copy number alterations cover large sections of each chromosome (Chr) across the genome. **C:** There is large variation of copy number across the genome. **B** and **D:** BAF plot shows higher allelic imbalance (**D**) than in corresponding allelic-specific copy number profile plots (**B**).

The Allele-Specific Copy Number Analysis of Tumors package generates an allele-specific copy number profile for the sample across the entire genome. Briefly, both alleles are represented, with the allele having the highest copy number shown in red and the lowest copy number shown in green. In contrast, the B-allelic frequency plot displays the allelic copy ratio of the A and B genotypes without considering aneuploidy. In short, values of 0 and 1 represent genotypes A and B, respectively, whereas intermediate values represent mixtures of genotype AB, with 0.5 indicting no allelic imbalance in the copy ratio. A representation of this is shown in Figure 3, which presents two samples with positive and negative GIS scores. Figure 3, A and B, shows a GIS-negative sample. Although the allelic copy number varied across the genome, with a major disturbance in copy number observed in chromosomes 7, 8, and 11 (Figure 3A), the allelic frequency distribution remained primarily heterozygous with a normal allelic count of 0.5 (Figure 3B). Conversely, a GIS-positive sample showed an allele-specific copy number plot with changes in the characteristics of telomeric allelic imbalance, large-scale state transition, and loss of heterozygosity (Figure 3C). Similarly, the B-allelic frequency plot (Figure 3D) revealed an allelic imbalance as evidenced by changes in allelic copy ratio values.

### GIS Score Performance

**Figure 4:**
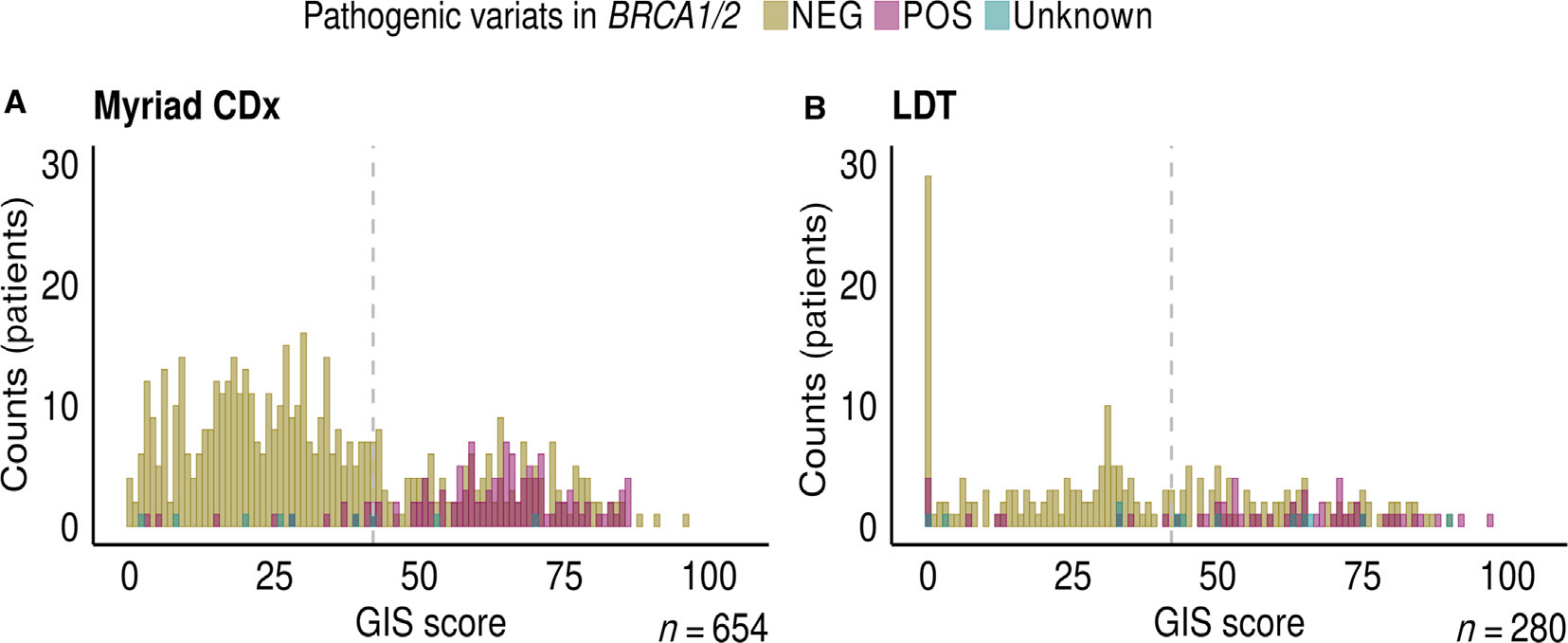
**A** and **B:** Genomic instability scar (GIS) scores and *BRCA1/2* mutation status of 654 samples analyzed externally by Myriad myChoice CDx (**A**) and 280 internally analyzed samples (**B**). Pathogenic *BRCA1/2* variants represented as in violet, nonpathogenic or no variants in green, and unknown *BRCA1/2* status in blue. **Dashed line** represents the GIS threshold value of 42 at which a sample is considered GIS positive (POS; ≥42) or GIS negative (NEG; <42). **A:** Myriad GIS-positive samples correspond to 41.4% (271/654) from which 41.3% (112/271) harbor *BRCA1/2* pathogenic variants. **B:** In a similar manner, laboratory-developed test GIS-positive samples correspond to 48.4% (136/280) from which 33.1% (45/136) harbor pathogenic *BRCA1/2* variants.

The GIS scores and *BRCA1/2* variant classification statuses from 654 samples analyzed using Myriad myChoice CDx were compared with those 280 internal research samples, where the GIS scores and *BRCA1/2* variant classification statuses were evaluated locally (LDT) (Figure 4). Overall, 271 of 654 Myriad samples (42%) and 136 of 280 LDT samples (49%) had positive GIS scores (score ≥42). Among the Myriad analysis results, 10 of 383 GIS-negative samples (2.6%) and 112 of 271 GIS-positive samples (41.3%) harbored *BRCA1/2* pathogenic variants according to the Myriad criteria. In the LDT analysis results, 10 of 144 GIS-negative samples (6.9%) and 45 of 136 GIS-positive samples (33.1%) harbored pathogenic *BRCA1/2* pathogenic variants, which were classified as such, by the VarSome^15^ annotation platform (VarSome, *https://varsome.com*, last accessed September 20, 2024). More detailed information on the numbers and percentages of GIS/*BRCA1/2* cases can be found in Table 2.

**Table 2:** Comparison between Samples Analyzed by Myriad or by LDT

Variable	Myriad myChoice CDx				LDT			
	*N*	% Total	% From analyzed samples	% Based on GIS	*N*	% Total	% From analyzed samples	% Based on GIS
Total	730				286			
Samples with GIS score	654	89.59			280	97.90		
GIS score ≥42	271	37.12	41.4		136	47.55	48.4	
Pathogenic *BRCA1/2* variants								
Yes	112	15.34	17.1	41.3	45	15.73	16.1	33.1
No	157	21.51	24.0	57.9	83	29.02	29.6	61.0
Unknown	2	0.27	0.3	0.7	8	2.80	2.9	5.9
GIS score <42	383	52.47	58.6		144	50.35	51.4	
Pathogenic *BRCA1/2* variants								
Yes	10	1.37	1.5	2.6	10	3.50	3.6	6.9
No	366	50.14	56.00	95.6	130	45.45	46.4	90.3
Unknown	7	0.96	1.10	1.8	4	1.40	1.4	2.8
No GIS score	62	8.49			6	2.10		

GIS, genomic instability scar; LDT, laboratory-developed test.

Table 3 describes the types of pathogenic variants present in the GIS-negative, *BRCA1/2*-positive samples. In the Myriad analysis, the *BRCA1/2* variants identified in the GIS-negative samples were predominantly frameshift mutations (8 of 10). In contrast, missense variant *BRCA1* [NM_007294.4 (VarSome, *https://varsome.com*, last accessed June 7, 2024) c.181T > G (p.Cys61Gly)] was the most commonly observed variant in the LDT analysis (4 of 10). A list of all identified *BRCA1/2* variants can be found in Supplemental Table S1.

**Table 3:** *BRCA1/2* Variant Types Found in GIS-Negative Samples

*BRCA1/2* variant type	Samples: Myriad, *N*	Samples: LDT, *N*
Frame shift (deletion)	8	0
Frame shift (insertion)	0	1
Missense Cys61Gly	1	4
Other missense	0	3
Nonsense	0	1
Noncoding	1	0
In-frame deletion	0	1

GIS, genomic instability scar; LDT, laboratory-developed test.

### Clinical Treatment Decisions

Treatment decisions were collected from a cohort of 90 patients, 49 of whom had a positive HRD score (≥42) by Myriad or LDT. Among these patients, 6 were exclusively recommended a PBC, 17 were recommended niraparib in combination with PBC (PBC/niraparib), and 26 were recommended olaparib in combination with PBC. Within the subset of 26 patients recommended PBC/olaparib therapy, 12 were prescribed olaparib as a PARP inhibitor based solely on their positive GIS status, whereas the remaining 14 also harbored additional *BRCA1/2* pathogenic variants.

These analyses revealed that determining GIS scores led to an 85% (12/14) increase in the number of patients who may benefit from olaparib as a PARP inhibitor therapy.

### Online Platform: openHRD

The online platform openHRD can determine the HRD based on the GIS score. It is freely accessible via the following link (*https://dga.medunigraz.at/hrd*, last accessed March 24, 2025).

To calculate HRD, the user requires the raw OncoScan CEL files and sample tumor content. In the first step, the CEL files are uploaded to the platform and the analysis is run. Once the results are ready, the page provides several graphs, including the B-allele frequency score graph, sunrise plot that indicates the best-determined tumor content and ploidy in the sample, and allele-specific copy number profile. The user must confirm that the tumor content aligns with that prediction made by the pathologist. If the algorithm cannot determine tumor content or ploidy accurately, manual adjustment of these parameters is available for re-analysis.

The results section also includes quality metrics, to validate the accuracy of the copy number calls with the median of the absolute values of all pairwise differences (accepted ≤0.30) and the control of diploid markers with the single-nucleotide polymorphism quality control of normal diploid markers (accepted ≥26; OncoScan Console 1.3 User Guide, per manufacturer’s instructions). Finally, the individual GIS scores are presented in a table format with the total GIS score, both with and without correction.

All results can be downloaded as a zip file for the user, and no information is stored.

### Data Availability Statement

All necessary information has been included in the article. Raw data acquired in the LDT assay are available on request.

## Discussion

Considering the availability and effectiveness of PARP inhibitor treatments for patients with ovarian cancer with a homologous recombination deficiency phenotype, reliable and rapid HRD-GIS determination methods are clearly needed.

By the end of 2022, Myriad myChoice CDx was established as the gold standard for HRD determination. However, it has several disadvantages, including high costs, long turnaround time, and the requirement for samples to be sent outside the European Union jurisdiction.

Several research groups have demonstrated that HRD-GIS score determination can be performed locally in academic laboratories.^19^, ^20^, ^21^ The present results show that HRD determination based on GIS using the LDT method has a high concordance with the GIS status of the compared Myriad myChoice CDx assay, with a sensitivity of 93% and a specificity of 95%. The overall percentage agreement of 93.6% with the LDT is similar to the 93.1% obtained by Tsantikidi et al^22^ with their proposed pipeline and higher than the 77.5% obtained by Fountzilas et al^23^ with the OncoScan platform, the 87.8% obtained by Fumagalli et al,^19^ and the 83.3% obtained by Fountzilas et al^23^ with the AmoyDx kit. Although some false-positive and false-negative results were obtained, the sensitivity and specificity of the LDT assay above 90% gives confidence to the results.

When reviewing analysis performance, the internal GIS score analysis had a higher success rate (97.9%) compared with the Myriad myChoice CDx analysis (89.6%) and the AmoyDx kit analyses (84%).^19^ The higher sample quality requirements of the Myriad myChoice CDx may account for this difference. The internal analysis prioritizes tumor content and DNA concentration when deciding to reject samples. Notably, the Myriad myChoice CDx documentation states that it can work with a DNA amount as low as 30 ng (MyChoice CDx Technical Information, per manufacturer’s instructions), and studies suggest that 30 ng of DNA is sufficient for valid microarray data.^24^ However, all internal samples processed in this study contained 80 ng DNA, as recommended by the manufacturer (Thermo Fisher Scientific), ensuring the efficacy of the DNA hybridization process.

A limitation of this study is the number of samples available for the training and validation groups of the LDT; unfortunately, there was insufficient material from additional Myriad samples to extract DNA and perform LDT analysis for comparison.

The *BRCA1/2* distribution was similar between the internal LDT and Myriad myChoice CDx analyses, with most of *BRCA1/2*-negative samples found in the GIS-negative group and approximately 50% of samples in the GIS-positive group harboring *BRCA1/2* pathogenic variants. These findings align with the observations made by other groups, where the HRD phenotype is not exclusively related to the presence of *BRCA1/2* variants.^25^^,^^26^

This study found that HRD determination significantly aided in guiding treatment decisions regarding PARP inhibitor therapy. Specifically, the GIS assessment expanded the cohort of patients eligible for olaparib treatment, irrespective of the *BRCA1/2* variant status. This is beneficial because the use of olaparib has been shown to increase overall survival, as demonstrated in the PAOLA-1/European Network for Gynaecological Oncological Trial groups (ENGOT)-ov25 trial^27^; without a positive HRD status, this treatment cannot be reimbursed to patients.

Several research groups have developed their own pipelines for HRD-GIS determination, often requiring users to pay fees. Examples include the GSA by Chen et al^28^ and RediScore by Tsantikidi et al.^22^ In contrast, the openHRD platform offers a free alternative, allowing users to calculate GIS scores and determine HRD using only CEL files as input.

Despite the valuable results from internal GIS score analysis, implementing this analysis in a diagnostic setting is challenging because of the proprietary software and patent-protected GIS values for diagnostic use. Genomic scarring cannot always correctly predict a good response to PARP inhibitor treatment because of the potential regain of proficiency in the homologous recombination repair mechanism.^29^ Therefore, complementary functional studies that provide real-time information about the HRD status may be beneficial for determining HRD more effectively.^4^

In conclusion, this study validated the use of the OncoScan CNV assay in combination with accessible analytical packages, providing a reliable method for HRD-GIS determination that can be performed in properly equipped molecular biology laboratories on a large-scale sample cohort.

Although next-generation sequencing technologies can generate similar results with concurrent information about *BRCA1/2* variants and the status of BRCAness HRD genes, no open- or cross-platform next-generation sequencing assay for HRD GIS is currently available. Additionally, the OncoScan Microarray protocol has been optimized for FFPE tumor samples in which the nucleic acids are often modified and degraded. The protocol established in this study allows for cost-effective, rapid, and reliable GIS scoring of FFPE samples and would be a valuable addition to the diagnostic workflow for ovarian cancer treatment.

## Disclosure Statement

None declared.
